# Collectanea

**Published:** 1830-04

**Authors:** 


### COLLECTANEA.

Floriferis ut apes in saltibus omnia libant,
Omnia nos, itidem, depascimur aurea dicta.

### ANATOMY.

*Rudiments of a Fœtus found in the Testicle of an Infant six months old.* By M. J. Wendt.—The wife of a blacksmith in a village near Glogau was delivered of a male child in December 1817. The infant was well made, and healthy. In May 1818 it came under the care of a surgeon, who discovered a hard tumor of the right testicle, with a congenital phymosis, which was cured by an operation. In the month of June, the diseased testicle had become so large as to descend quite to the knees. The tumor was cold, unequal, hard, and very sensible to the touch. It was removed by an operation in July; and the child, without the occurrence of any unpleasant symptom, was perfectly restored to health in less than a month. The extirpated testicle weighed about seven ounces. Its parenchyma was filled with an oily, ichorous, or yellow fluid. The epididymis was entirely wanting. When the tunica vaginalis was opened, a hard body was discovered, which proved to be a thigh bone deprived of periosteum. The bones of an imperfect pelvis were found along with it, connected together, and mingled with cellular tissue and muscular fibres. The rudiments of the lumbar vertebræ, and a lower extremity composed of skin, muscles, and bones, but exceedingly deformed, were also recognised. The other parts of the body appear to have been wanting.—*Journal Universel des Sc. Med.*

### PHYSIOLOGY.

*Proportion between the Nervous System and other Parts.* M. Tiedemann has reported several cases of defective developement of the spinal marrow, as correspondent to a congenital absence of the limbs; and, on the other hand, cases of excessive developement of the brain and nerves having relation to the existence of supernumerary organs. M. T. regards these phenomena as constantly observable in such cases. The Professor concludes that the nervous system, as being the first existing part of the animal, regulates the formation and ulterior developement of the embryo, and determines the particular form and disposition of the other organs.—*Archives.*

*Sense of Touch.* The presence, in insects, of the ganglion which represents the brain, is not absolutely necessary for the existence of the sense of touch. After decapitation, they feel, on the surface and in their limbs, by means of their other ganglions, such impressions as may be made on them. The spinal marrow of reptiles, young birds, and young mammiferous animals, seems also capable, after the abolition of the brain, of being modified by irritations, of feeling them, and of occasioning, in consequence thereof, durable and calculated movements, which are not to be confounded with those convulsive and fugacious motions that are attributable solely to irritability. M. Calmeil thinks that this faculty of the spinal marrow is probably diffused throughout its whole extent. Further, it is probable that, in the natural state of our functions, the brain is the sole centre of irritability, and that the spinal marrow only becomes sensible when the brain ceases to exist. The co-ordination of our voluntary motions is doubtless attributable only to the brain.

---

### PATHOLOGY.

*Hemiplegia of Sensation, without Loss of Motion.* By M. LE SAUVAGE, of Caen. (*Bull. des Sc. Med.*)

Notwithstanding the multiplied researches of modern physiologists, and the ingenious experiments which have been devised to explain the mechanism of the functions of the nervous system, we are yet unable to determine what parts of the encephalon are the exclusive seats of sensibility, or voluntary motion. But few facts similar to the following are on record.

M. Loriot, seventy-three years of age, had always enjoyed good health. For a few days he had felt occasional giddiness, when, on the 10th March, he suddenly experienced a numbness in the whole of the left inferior extremity. It appeared to him as if his foot was sinking into the earth, and he held his thigh firmly with his hands, to prevent himself from pressing so heavily upon the ground. Almost at the same instant the numbness extended over the whole of the left side of the body. He hastened home in much alarm, and walked without difficulty. The next day he was in the following state: Intellectual faculties unimpaired; pulse unaffected. He could walk and move his arms as usual, excepting that the elevation of the arm by means of the deltoid was somewhat limited. He was not conscious of the movements he executed, nor of the impression of bodies which he touched. The skin of the whole of the left side of the body was perfectly insensible: it was pinched and pricked violently without producing pain, or indeed any sensation at all. On the anterior part of the body the median line was not the precise limit of the sensible parts. On the left side the skin was still easily sensitive to the extent of about an inch beyond the central division, but beyond this point it was perfectly insensible. On the affected side neither the sense of sight nor hearing was altered, but that of smell and taste was abolished. If the left side of the tongue was moistened with strong vinegar, the patient was unconscious of the slightest impression, whilst the flavor of the liquid was keenly perceptible by the other half of the organ. Strong odours placed under the nostril of the affected side were not perceived, excepting when M. L. drew a deep inspiration; but then the odoriferous molecules arrived at the right nostril by the posterior opening of the nasal fossa. When M. Sauvage placed his hand across the patient's head, that part only of it was felt which pressed upon the right half of the head. Every treatment that was suggested was ineffectual.

A few months after the attack, M. L. had resumed his ordinary occupations. For some time he felt occasionally dull transient pains on the hemiplegic side. He could move the limbs of that side without difficulty, but still he felt their motive powers were inferior in degree to those of the other side. In other respects the constitution of the patient had not apparently suffered.

---

In the third volume of the Medico-Chirurgical Transactions, Dr. YELLOLY relates a somewhat similar case. The patient, a man, after being much heated and fatigued, slept with the window open. In the morning his feet and ankles were numb, without pain, and without the muscular power being at all affected. A tingling in the little finger afterwards occurred, and gradually both hands were in a considerable degree insensible. The hands up to the wrists, and the feet half-way up to the leg, were perfectly insensible to any species of injury, as cutting, burning, &c. The insensibility did not suddenly terminate; it existed to a certain degree nearly up to the elbow, and for some distance above the knee. He accidentally put one of his feet into boiling water, but was no otherwise affected by it, or aware of the high temperature, than by finding the whole surface a complete blister on removing it. Extremities insensible to electricity. His skin, in general, healed very readily after being burnt or scalded. The existence of muscular power, and the faculty of directing its exercise by the will, remarks Dr. Yelloly, where the nerves have entirely lost that sensibility which is always regarded as necessary to the conveyance of volition from the sensorium, are circumstances apparently irreconcileable with any knowledge which we at present possess of the mechanism by which the will acts in the production of voluntary motion.* Thanks, however, to the investigations of modern physiologists, we are *now* capable of understanding how it is that muscular power may be preserved whilst sensation is lost, or vice versâ. If the anterior fasciculus of the spinal nerves be affected, motion will be impaired, and sensation remain; if the posterior fasciculi be affected, sensation will be impaired, while motion will remain. The above examples, then, though curious from their infrequency, can no longer be regarded as pathological wonders, but as interesting illustrations of the correctness of the opinions of modern physiologists.

EDITOR.

---

*Spontaneous Combustion of both Hands.* The following case of spontaneous combustion, from the *Archives gén. de Médecine* for March 1829, is superior in point of authenticity to any yet recorded.

A gentleman, of a robust, healthy constitution and temperate habits, twenty-four years of age, extinguished with his hands the burning clothes of his brother, who had accidentally set fire to them with sulphur, and was immediately afterwards attacked with such acute pain in both hands as to compel him to cry out for assistance. A woman, who came to his succour, observed that both hands were surrounded by a blue flame. This at first was supposed to be occasioned by the sulphur adhering to them, and an attempt was made to extinguish the flame with cold water, but without effect. The gentleman then ran down stairs to a cutler's shop, and plunged his hands into a quantity of mud: from this he derived very little relief. After suffering in this manner much torture for half an hour, he ran to the house of Dr. Richond de Brus, by whom the case is related. On the way, both he himself and the

* GALEN speculated upon this subject in the following manner: Motion, he says, is *active*, sensation *passive*, and therefore a greater nervous power is necessary for the former than for the latter. There may, then, be nervous power sufficient for sensation, though not sufficient for motion; so that sensation may remain, though motion be lost. With respect to those cases in which motion remains, sensation being lost, he observes that motion is performed by nerves through the medium of muscles, but that sensation depends upon nerves distributed to the skin. Now, the nerves of the skin may be injured, and those of the muscles uninjured, and thus motion may remain though sensation be lost. When the limbs are all paralysed, the common principle is affected, and both sensation and motion are destroyed together.—EDITOR.

woman who accompanied him observed distinctly the blue flame surrounding the hands. The physician met him at the door, and observed the hands to be red, swelled, and exhaling a kind of smoke or vapor.

He immediately directed his patient to plunge his hands into a well which was opposite, and to keep them there until he experienced relief. On his doing so, the pain abated considerably, and the flame ceased; but he had not gone more than 150 paces homewards, when they reappeared. On reaching his dwelling, he immersed each of his hands in a bucket of water, which, as it got rapidly heated, he had repeatedly renewed. As often as he took them out of the water, he remarked a sort of unctuous matter flow along his fingers, and the blue flame reappeared: the latter was not however visible, except in a situation where the light of the candle was shaded, as under the table. A young gentleman, who remained in the room with him, saw the blue flame several times in the course of the night. Towards daybreak, sparks only were visible. During the succeeding day the pain was very severe; and large vesications, filled with a reddish serum, had formed on the fingers: in some places, indeed, the cuticle was entirely removed, and the cutis grayish and corroded. The vesications being opened, cerate was applied to the denuded surfaces, and the whole covered with poultices. The inflammation which followed was moderate, the suppuration healthy, and in six weeks the ulcers caused by the burning were entirely healed; but the cicatrices were very distinct, and several of the nails had dropped off.

If, observes the relater, the flame had been perceived only immediately after the burning of his brother's clothes, it might have been supposed to have resulted from some portions of burning sulphur adhering to the skin. But it resisted the effects of repeated cold affusion, and of protracted immersion in cold water; it continued all night, and was spontaneously reproduced after having been extinguished in the water of the well. It was at first so bright as to astonish those who witnessed it, and to cause the female who first saw it to say that the hands burnt like candles. Such an explanation, therefore, is quite untenable. Although, when the patient first reached my house, I only saw a kind of smoke, yet it must be remarked that I had a candle in my hand at the time, and my servant another, so that the pale light emitted by the hands may have been then present, but eclipsed by the superior light of the candles. If it was more visible on his return home, this might clearly have arisen from its being brighter, as well as the apartment darker. To what, then, should this combustion be ascribed? To hydrogen gas? But how could this gas have been generated? We admit, as probable, that the brisk excitement occasioned by the first burning caused the disengagement of phosphureted hydrogen gas, which would inflame on coming in contact with the air. Thus might be explained the trivial nature of the burns. But could we also account in this manner for the burning pain felt by the patient all the night after, for the continuance of the discharged gas, and the reproduction of the extinguished flame? Electricity does not seem to me to explain the matter any better.

The case is very analogous to one which is related in the Nouv. Journal de Medecine for December 1822.

---

*Suspension of the Cerebral Functions from Irritation of the Stomach.* By Dr. G. B. Wood. (Extract from the *North American Med. and Surg. Journal.*)

A partial suspension of the cerebral functions is well known to be an occasional attendant upon irritation in the stomach. In some instances the symptoms so closely resemble those of diseased brain, that the physician is in some danger of mistaking the real seat of the disorder, and consequently of misapplying his remedies. We may, however, generally form a correct judgment from the preceding history of the case; and, when this cannot be ascertained, may often satisfy ourselves by observing the effects of pressure upon the epigastrium.

A young man, subject to dyspepsia, was suddenly attacked, about an hour after his evening meal, with an affection supposed to be apoplectic. When I arrived, he was lying on his back, without motion, wholly unable to see or hear, and showing no sign of sensibility, even when pretty smartly shaken. He had not fainted; for the respiration and circulation continued; and the absence of stertor, with the natural condition of the pulse, was incompatible with the notion that the disease was apoplexy. Having learned that he had eaten cucumbers and whortleberries at his tea, and that he had afterwards complained of headach with some nausea, I had little doubt that the root of the evil would be found in the stomach. An emetic was accordingly administered, which, after a much longer interval than usual, operated freely, and brought away the undigested berries and cucumbers, which were undoubtedly the cause of irritation. Sensibility was now speedily restored; and, after an attack of most violent spasm in the bowels, which was relieved by laudanum and castor oil, the patient recovered his usual health. He had never before been affected in a similar manner.

Little more than a month has passed since I was requested to visit a gentleman said to be in a dying state. I found him apparently without sensation, his eyes open and turned up, his hands clenched, and his body alternately motionless and agitated by sudden and universal tremors, which caused the bed to shake beneath him. He was a stranger, and there was no one present who could give me a history of his case. In order to explore into its nature, I placed my hand upon his epigastrium; but scarcely had I touched the skin, when he started up as though a bullet had been driven through him. Uncertain whether the coincidence might not be accidental, I repeated the experiment several times, and at each time the slightest pressure was sufficient to throw the whole frame into immediate and violent, though brief, convulsions. Sufficient evidence was thus afforded of the seat of the disease, but not of the precise nature of the irritation. As his pulse was active, I bled him freely, and immediately afterwards applied a large sinapism over the region of his stomach. Consciousness was so far restored a few minutes after the bleeding, that, upon being asked in a loud voice if he felt sickness or pain in the stomach, he nodded in the affirmative. An injection of assafœtida was now administered; and, under the united influence of this remedy and the mustard plaster, he revived to some knowledge of his situation, and was able to drink very freely of warm water, which I urged upon him. This soon produced the discharge of a considerable quantity of acid liquors from his stomach, and restored him for a time to complete consciousness. He now told me that he was subject to gout, of which he had recently had an attack in his foot, but had relieved himself by bathing the affected part with hot vinegar. The nature of the case was evident. While he was yet speaking, he was seized with a sudden spasm of the stomach, which threw him into his former state; and this alternation of consciousness and insensibility was repeated several times within the course of a few minutes, each return of pain being so severe as at first to throw the whole nervous system into violent agitation, and then to overwhelm it for a time in complete torpor. I now applied sinapisms to the feet, and gave a mixture of laudanum and the aromatic spirits of ammonia; and, at the end of about four hours from the commencement of the attack, left my patient very greatly relieved. A dose of the compound tincture of rhubarb, with a proper regulation of the diet, was afterwards sufficient to complete the cure.

---

*Case of Paralysis occurring in a Child, and attended with anomalous Symptoms.* By J. Marshall Paul, m.d. of Philadelphia. *(Ibid.)*

An anomalous form of disease occurred in a little girl at the age of three years, and continued to affect her in a greater or less degree until her death, which took place in her sixth year, at the time she was recovering from measles.

It first exhibited itself by a complete loss of power over the muscles of the back and inferior extremities, as she was riding in a carriage; and she remained in this condition for at least half an hour, during which time I saw her. She was bled several ounces from the arm; took castor oil to open the bowels, as they were costive; had frictions of different kinds applied to the spine and limbs; and in a very short time (about one hour from the period she was seized,) she had so far recovered her usual power over the affected parts, as to be able to walk about the room without assistance. This temporary affection returned the following morning, and continued to do so for several successive days. It was now more particularly confined to the right limb, and was relieved by the same remedies, with the exception of the bleeding, which did not appear to be indicated by the pulse. An interval of several weeks then elapsed, during which time the child enjoyed excellent health, and exhibited no symptom of disease whatever. At the end of this time, a recurrence of the paroxysms took place, affecting her in a similar manner and for the same length of time as in the first instance; and they then ceased, leaving her in the possession of apparent health. Thus she continued to be attacked at intervals more or less distant, though with great abatement in the violence of the disease, until the period of her death, without exhibiting any marked symptoms of a cerebral affection, if we except a slight twitching of the muscles of the eyes and face, of momentary duration, on one or two occasions, and a disposition to sleep, which was generally indulged, soon after the termination of each paroxysm. It is proper to state that there was evidently a predisposition to epilepsy in this child, accompanied by a scrofulous diathesis; as evinced by her whole appearance, and by great irritability of the nervous system. She was never able to articulate words distinctly, as all of them uttered by her were gutturals, and difficult to be understood by persons not accustomed to her manner of expressing her ideas.

She was of a very costive habit. Her appetite was generally good, and at times voracious. She had been much indulged in every variety of food, and this in large quantities. Her abdomen was mostly hard and distended; and the discharges procured by medicine from her bowels were always, at the periods of an attack, extremely vitiated and offensive.

The course of practice instituted was a total change in the child's regimen, confining her entirely to a mild vegetable and milk diet, and in moderate quantity. Gentle cathartics were frequently administered, with a view to correct the state of the bowels. Frictions to the spine and extremities were employed; also the salt bath; and for a time a discharge was kept up from the back of the neck by means of a perpetual blister. The chief dependence, however, was placed on the laxatives ; as the alimentary canal was considered to be the primary seat of the affection, although some of her symptoms might be suspected to be of cerebral origin. The attacks diminished both in frequency and force, and the disease appeared almost subdued by these remedies. Indeed, we felt strongly encouraged to indulge the hope of the patient's ultimate recovery; as the affection had become so slight towards the latter period of her life as scarcely to attract attention, or cause a momentary inconvenience to herself.

It is worthy of particular observation, under all the circumstances of this case, that the child had always been remarkably exempt from colic and other bowel affections; for it proves that extensive disease, or unnatural alteration of structure, may exist in the intestines without being indicated by any one symptom, or readily suspected by the physician. About the beginning of the present year, she took the measles, had them mildly, and bid fair to recover; when, on the morning of the day on which the last of the eruption disappeared, she was, according to her mother's account, seized with a fit, which, on inquiry, did not answer to the description of a convulsion. The child appeared greatly distressed, applied her hands to the stomach, screamed, and immediately became rigid in her limbs and body. By the time the neighbouring physician saw her, it had passed over, and she was as well as usual. Some medicines were directed, and he went away. Three or four hours afterwards, the fit returned with greater violence, and left her in a state of stupor and insensibility until her death, which took place about twelve hours afterwards.

An examination was made by Dr. J. Rodman Paul and myself, thirty-six hours after death. The brain presented no morbid appearance whatever: it was natural in size and texture, and uncommonly free from that turgescence of blood-vessels which is to be met with in most subjects. No effusion of any kind, either in the ventricles or between the membranes. No deviation from the natural appearance to be observed in the medulla oblongata. Stomach of the usual size, and its mucous coat healthy, except two or three small injected spots near the cardiac orifice. Liver, spleen, pancreas, and kidneys natural. In the small intestines we discovered four distinct intus-susceptions, each to the extent of four or five inches. The first was found at the distance of twenty-eight inches from the pylorus; the second, fifteen inches from the first; the third, about six inches further on; and the fourth occurred eight inches from the third; all bearing a striking resemblance to each other. The intus-suscepted parts were much thickened, and the caliber of the tube was considerably diminished. The muscular coat of the intestine was developed in a remarkable degree just at the contracted portions, as if nature had endeavoured to overcome the obstruction by giving it additional power. Every thing indicated that the disease was not of recent origin, but was probably coeval with the first appearance of her singular attacks; as we were struck with the unusual thinness of the parietes of the bowel above each intus-susception. The mesentery was studded with enlarged glands, of the size of a split pea. No other morbid affection was observed.

I am perfectly aware that the state of the bowels, as above described, is sometimes attributed to the free use of drastic purgatives; but in this case there is not sufficient ground for adopting such an explanation, as the medicines employed were mostly of a mild nature, and acted gently, without producing pain or inconvenience.

---

*Delirium Tremens.* In RUST'S Magazine for 1829, No. iii. Dr. SCHIRLITZ relates a fatal case of delirium tremens, with the appearance of the brain after death. It occurred in an habitual drunkard. The symptoms of the disease were well marked. The treatment consisted of the application of twelve leeches to the forehead and temples; of injections of cold water into the rectum twice a day; cold applications to the head; blisters to the nape of the neck; and every two hours one grain of calomel and one fourth of a grain of opium, with a spoonful of a mixture of infusion of Valerian and Liq. Ammon. Acet. between each dose. He died on the ninth day of the disease in "apoplectic convulsions." The skull being opened forty-eight hours after death, the dura mater was found inflamed; the vessels of the brain somewhat engorged with blood, the lateral ventricles containing about six ounces of a clear fluid. The examination was not permitted to be carried any further.

---

*Compression of the Diaphragmatic and Pneumo-gastric Nerves simulating Disease of the Heart.* A female, sixty years of age, had for many years experienced a pain behind the sternum and at the base of the chest, which occurred in paroxysms. This pain, which was at first slight, augmented in intensity, preserving however its paroxysmal character, and became soon accompanied with violent palpitations of the heart, difficulty of respiration, œdema of the upper extremities, and a small pulse: in a word, with all the symptoms indicative of an obstruction to the circulation through the superior extremities. This patient remained for two years at La Pitié, during which time her chief complaints were of the pains behind the sternum, and at the lower part of the chest. For a long time she had no appetite; emaciation was extreme, and her voice was remarkably weak. The patient finally died.

On examining the body, it was discovered that the pneumo-gastric and left diaphragmatic nerves, the aorta and its branches, and the veins of the superior extremities, were surrounded in different parts of their extent by masses of a scirrhous nature, by which they were compressed, but not disorganized. The heart, which preserved its ordinary dimensions, was of a violent colour, much deeper than ordinary; its tissues also appeared softened. The lungs were healthy, with the exception of the right, which presented at its summit an excavation filled with blackish blood. In the digestive organs there was observed the commencement of atrophy; otherwise they were healthy.—*Bibliothèque Médicale.*

---

### PRACTICAL MEDICINE.

*Intermittent Fever cured by Levigated Charcoal.* The *Journal des Progres* gives three cases of intermittent fever, which Dr. PŒRQUIN cured by levigated charcoal, in doses of two drachms, mixed with water or some pleasant vehicle. A single dose seems to have been successful in each of the three cases.

---

*Treatment of Hooping Cough.* (*Journal der Practischen Heilkunde.*)

Dr. RAHLEISS, who published in 1827, in Horn's Archives, a memoir upon the efficacy of the following remedies in hooping cough, after having employed them with the greatest success in more than a hundred cases, has inserted in Hufeland's Journal another article upon the same subject, for the purpose of strengthening his previous observations by his subsequent experience. The following is the form of the remedies advised.

℞. Pulv. Belladonnæ gr. iv.

—— Doveri gr. x.

Flor. Sulph. loti ℈iv.

Pulv. Sacch. alb. ʒij. M. et div. in ch. xx.

Dose for an infant two years old, one of these powders every three hours. Between each dose, a teaspoonful of the following mixture is to be given.

℞. Infusi Anthem. ℥i.

Syrupi ʒij.

Acid. Hydrocyanici (Vauquelin) gtts. xij. M.

The proportion of the powerful ingredients which enter into these formulæ may be augmented or diminished according to the age and temperament of the child.

In concluding his memoir, M. K. observes, that sometimes the effects of these remedies are not perceptible for five or six days, but that they then become very manifest, and that, generally, in eight or twelve days at most the cure is complete. In some cases, after two or three days' employment of the remedies, an efflorescence upon the skin is perceived, and some dilatation of the pupils. The treatment must be then suspended for twenty-four or thirty-six hours, and the quantity of the belladonna diminished.

⁂ Of the above combination of powerful remedies we have no experience. We have frequently tried the prussic acid in hooping cough, without any apparent advantage.—Editor.

---

### SURGERY.

*Efficacy of Lemon Juice in some Diseases of the Eyes.* (From the *Journal für Chirurgie und Augenheilk.* t. xiii. cah. 2, p. 224, 1829.)

M. Werlitz thus employs this novel remedy. He cuts a slice of lemon-peel about an inch long, and half an inch broad, places the outer part opposite the affected eye, the eyelids being opened, and by slight pressure squeezes out the little drops of volatile oil contained in the tissue of the rind into the eye. The sensation produced is acute, and continues for an hour or two. If the pain caused should be severe, cold applications are to be employed. The oil of the lemon-peel appears to increase the capillary circulation, and to cause the absorption of morbid depositions.

From experiments which have been made at La Charité at Berlin, it appears that the following diseases are remedied by this treatment: 1. Inflammations of the eye which are passing into a chronic state, and which affect the external parts, as the conjunctiva, cornea, or sclerotic, particularly if the small vessels are turgid. M. W. has also found the remedy useful in rheumatic, gonorrhœal, and scrofulous ophthalmies. 2. In pannus and pterygium. 3. In albugo, and opacity of the cornea. 4. When the texture of the cornea has lost its healthy density, and becomes soft and spongy. The remedy may be employed frequently during the day, depending upon the degree of irritation it produces. M. W. relates seven cases of cures of various diseases of the eye affected by this treatment.

*Case of severe lacerated Wound of the Rectum and Bladder.* By CHARLES HALL, M.D. of St. Alban's, Vermont.

In the month of May, 1828, I visited Charles B. Weston, of Sheldon, an industrious and respectable farmer, between fifty and sixty years of age. He had just received a most severe laceration of the rectum and bladder, occasioned by being brought to the ground with some degree of force, partially suspended by a slim staddle, which he had climbed, and by his weight had bent over, while attempting to destroy a nest of young crows, situated on a large tree standing near by. In this predicament he came to the earth, holding by his hands to the top of the small tree, his posteriors coming upon a dry beach bush, the body of which, the size of a large walkingstaff, being broken by the fall, passed, per anum, about ten inches into the abdomen, when his feet touching the ground, prevented its further progress. From this unpleasant position he with some difficulty extricated himself; and, on withdrawing the stub, his bladder emptied itself through the opening. He was brought to his house, where a few hours afterwards I saw him, in company with Dr. JUDSON, his family physician. There was no external laceration, the stick having passed in the natural course about two inches, where it perforated the rectum, and pierced obliquely upwards through the coats of the bladder. We were enabled to trace its course thus far with the finger. We could detect no foreign substance in this extensive wound; though, from the appearance of the broken and uneven end of the stub, we were led to suspect that some pieces of it had been left: this eventually proved, however, not to be the case. The unhappy patient experienced the most excruciating agony, and seemed to be in a hopeless condition. But nature, with the assistance of a few remedial agents, such as bloodletting, &c., performed a cure. For the first three or four days, his urine passed mostly through the wound; to prevent which, as well as to restore its natural course, recourse was had to the catheter. This, with the aid of other auxiliaries, soon restored the natural outlet, and the lacerated integuments gradually closed.

I am principally indebted to my friend Dr. Judson for the foregoing history of the case, for I saw the patient but once. The Doctor informs me that the man has recovered his accustomed health.—*American Journal of Med. Sciences.*

---

*Datura Stramonium in Retention of Urine.* By WM. M. FAHNESTOCK, M.D.

The season is now rapidly approaching when persons of advanced age are very liable to retention of urine, from exposure to cold and dampness, occasioning enlargement of the prostate gland, and muscular contraction of the membranous part of the urethra; and these frequently not only produce much inconvenience to the patient, but often very considerable embarrassment to the surgeon. The great sensibility of the parts, and their peculiar conformation, render it very difficult to overcome the obstruction in the diseased state. Much of the obscurity in these cases, however, arises from the want of a perfect knowledge of the minute anatomical structure, and an accurate acquaintance with the pathological state of the gland; and particularly the augmentation of the third lobe, which presents the chief difficulty in introducing the catheter; and which, by rashness, in pursuing the plan recommended by DESAULT, of pushing the catheter forcibly onward into the bladder, is frequently so extensively injured as to become the seat of permanent irritation, form a chronic enlargement, and prove an insurmountable barrier to all further efforts to restore it. Dr. Physick has very ingeniously contrived a bougie-pointed catheter, which can often be insinuated when other instruments cannot be passed; but even this is not practicable at all times.

Baffled in some inveterate cases which had sustained injury by injudicious treatment, we were led to try some relaxing medications to subdue the rigidity of the parts, and have succeeded so fully in a few cases with the stramonium, that we feel anxious to recommend it to the attention of the profession.

In the fall of 1825, we were called to see P. B., ætat. seventy-four, who by exposure to cold and wet had been suffering some days with retention of urine arising from an enlargement of the prostate gland. A variety of applications had been made, as emollients, demulcents, fomentations, ice, &c.; and great irritation had been excited by ineffectual attempts to introduce the catheter. The third lobe of the gland had been partially pierced, and become very tender: the least touch or pressure of the instrument would rupture its engorged vessels, and discharge profuse quantities of blood. The catheter was tried, but was arrested at the prostate gland; and being foiled in all our attempts with a variety of instruments, and in different positions, we ordered a large cataplasm of the leaves of the datura stramonium, and continued them three hours, after which we readily passed the catheter, and drew off a large quantity of urine, mixed with a dark grumous fluid. The following day we encountered the same difficulty in the introduction of the instrument, but which yielded again in a few hours after the renewal of the stramonium. The catheter was now allowed to remain two days in the passage, but excited so much pain and irritation as to oblige us to remove it before we could subdue the disease, and were again reduced to our former dilemma: by persevering, however, with constant applications of the poultices, the disease was entirely removed, and has not since returned.

Other similar cases have come under our observation, in the more advanced season, when the leaves could not be procured: under these circumstances we found a bath made of the seeds succeed admirably well. Cases attended with much pain and tenderness are very much relieved by bloodletting, and particularly by the application of leeches to the perineum.

Might not the extract of belladonna applied daily in cases of chronic enlargement, prove beneficial; or should we not expect an equally energetic operation from the Nicotiana tabacum? This might be tried in cases of emergency.—*Ibid.*

---

*On the Removal of Diseased Joints, instead of amputating the Limb.* By M. Roux. *(Revue Medicale.)*

From a paper lately presented to the Academy of Sciences by M. Roux, we extract the following remarks.

M. Roux deprecates the removal of the knee-joint in cases of white swelling or other diseases. The articulation is too extensive, and its removal causes the greatest constitutional disturbance. At the express desire of a patient, he once performed the operation, but death took place on the nineteenth day. He is of opinion that, even if the life of the patient could be preserved after such an operation, the leg would be more cumbersome and useless than an artificial limb. But he strongly recommends the removal of the elbow-joint, in preference to amputation, provided the hand can be saved. At this joint he considers the operation especially advantageous, and he is astonished that many surgeons should still prefer the amputation of the arm. By removal of the elbow-joint, which he confesses is, if not a difficult, at least a laborious task, M. Roux implies the removal of the whole of the inferior extremity of the humerus, and the upper extremity of each bone of the fore-arm. This operation will occupy from fifteen to twenty minutes. A very extensive wound is left, and, whatever precautions may be taken, abundant suppuration must follow, and a complete cure cannot be expected for several months. But if the limb be preserved, and restored to its functions, and if the life of the patient is not more endangered than by amputation, these inconveniences are amply compensated.

M. Roux has removed the elbow-joint four times; once in the right, and three times in the left arm. Three of the patients were men of a middle age; the fourth was a girl nineteen years old. In all, the disease of the joint, which was probably of scrofulous origin, was of a very formidable kind. There were numerous fistulous openings around the articulation, which was excessively swollen, and the operation afforded an opportunity of observing the complete fungoid degeneration of the cellular tissue, and the alteration in the structure of the bones at their articular extremities. One of these cases terminated fatally. In the other instances the cure went on favorably, but slowly; the lives of the patients were never endangered, and they regained a partial use of the limb. The young woman resumed her business as a milliner, and one of the men afterwards carried on the occupation of a grinder. The other died of consumption, with which he had been probably affected previous to the operation.

---

*Polypus of the Vagina removed by the local Application of Laudanum.* In HUFELAND and OSSAN's *Journal der Pract. Heilkunde,* February 1829, Dr. KAHLEISS, of Gröbzig, relates this case.

A woman stated that, during three or four months, she had been subject to a discharge of blood from the vagina, continuing during the intervals of menstruation. About four weeks previously, it had increased considerably, and had greatly weakened her. The discharge was unattended with pain. Various tonic and astringent remedies were tried, without benefit. The hemorrhage increased. The patient would not submit to an examination, and was not seen by Dr. K. for eight weeks. She then returned, and informed him that, four weeks before, she had felt something pass out from the vagina suddenly, and with perceptible noise; and that, in consequence, she now experienced some inconvenience in walking. On examination, a polypus was discovered, projecting two inches from within the labia. This had no doubt been forced down during the exertions of the patient, through the very narrow opening of the hymen, which still existed in full integrity. The polypus was of a deep red colour, bled upon the slightest touch, and had a lanciform shape.

Dr. K. having often succeeded in removing nebula and leucomata from the cornea by the repeated application of the liquid laudanum of Sydenham, and once in destroying completely, by the same means, a soft bloody excrescence on the head of a little girl, was induced to make trial of it in the present case. The polypus was well wetted with the laudanum twice a day. In twenty days it was so much reduced in size as to fall within the opening of the hymen. It being no longer within reach, a canula was passed down to it, and through this was introduced a camel's hair pencil, steeped in the laudanum. In this manner the whole of the polypus was completely destroyed by the end of the seventh month.

---

*Extirpation of the Parotid Gland.* We learn, from the *Nouv. Bib. Med.* for January 1829, that M. Fouillay, of Brest, has extirpated a tumor of the parotid gland, regarded as cancerous, from a female aged fifty-two years, which had become painful and interfered with mastication, deglutition, and even respiration. The carotid artery was secured by a ligature at the commencement of the operation; the division of the smaller vessels did not render ligatures necessary, excepting to the internal maxillary artery. A deep and irregular wound remained, which was dressed by means of sutures. No accident supervened. The ligature from the carotid artery came away in fifteen days, and the recovery was complete in seventy-five days.

M. Larrey suggests that there was no necessity for the ligature to the carotid artery, and that securing this vessel augmented the danger of the operation. In this opinion most of our American surgeons would probably unite, as they seldom resort to this measure in any of the important operations about the face or neck.—*North American Med. and Surg. Journal.*

---

*Extraction of a foreign Body from the Œsophagus.* Dr. J. B. Boileau succeeded in extracting a piece of copper money which was lodged in the œsophagus, about an inch below the pharynx, where it had remained eight hours, causing much pain, and severe but ineffectual efforts to vomit. The patient could with some difficulty swallow liquids and soft aliment. Dr. B. fixed a small conical portion of compressed sponge to the extremity of a piece of whalebone, and succeeded in passing the sponge thus secured into the stomach. Here he retained it, notwithstanding the patient's efforts to vomit, some minutes, until the sponge was distended by the fluids of the stomach. The instrument was now slowly withdrawn; the sponge completely occupied the canal of the œsophagus, and thus the foreign body was brought into the mouth with the sponge.—*Ib.*

---

### MATERIA MEDICA.

*On the Utility of Horseradish.* (*Journal Tchelovékolioubivago Obstochestva. Journal of the Philanthropic Society of Petersburg*, No. 2, p. 194.)

In Russia, the Cochlearia armoracia, horseradish, is highly esteemed in cases of suppression of the menses and chlorosis. In inveterate rheumatism it is also much resorted to; and is deemed preferable to mustard or cantharides for the purpose of stimulating the cellular tissue. It is infused either in wine, beer, or water. Two drachms of scraped horseradish are to be infused in about a pint of cold liquid, for twenty-four hours, in an ordinary temperature. The half or the whole of this quantity is to be taken in small cupsful in the course of the day.

*** Cullen, Bergiers, &c. have highly extolled this remedy; and, from our own experience, we are inclined to think it is too much neglected in modern practice. It is a powerful diuretic, and very useful rubefacient.—Ed.

### CHEMISTRY.

*Chemical Analysis of the Ergot of Rye.* By M. C. F. Maas, of Hamburgh. (*Kastner's Archiv. für dieges. Naturlehre*, t. xviii. p. 111.)

From the experiments of this chemist, it appears that the ergot of rye does not contain hydrocyanic acid, nor morphium, nor narcotine, as has been asserted. Ammonia is detected in it, or at least a vegetable alkaline substance, which perhaps is a peculiar alkaloid. It contains no phosphoric acid, but some acetic acid. Albumen, a violet colouring matter, and an alkaline residuum, are also constituents of the ergot of rye.

---

### LIMERICK MEDICAL SOCIETY.

At a recent meeting of this Society, the following interesting case was read by Mr. Wilkinson, which had fallen under his care in the County Infirmary.—The patient had a fistulous opening in the upper part of the left thigh, about two inches and a half below Poupart's ligament, and about one fourth of an inch to the inside of the femoral artery. A probe, passed into the opening, ran upwards and inwards as far as the pubis, for the length of about three inches. He said his urine had been passing through this opening for the last fortnight, and that it had done so for a similar period about twelve months ago. It flowed through the natural passage in the interval. He had marks of two old sores situated above the pubis, and separated by the linea alba, the effects of an abscess which formed about eight years since, and continued running for a long time, during which two small splinters of bone had come away. At the end of three years, an abscess presented at the upper part of the thigh, the site of the present fistula; on the bursting of which, urine was discharged. He kept a sponge constantly applied to the opening at the time of his admission. When recumbent, the water flowed through the urethra, but in the erect position it gushed through the fistula in a strong narrow stream. There never had been any obstruction to the natural passage of the urine, but matter was sometimes discharged by the urethra. He remained four days in the hospital, during which time the fistula closed, without any other attention than a dressing of adhesive plaster. The water passed afterwards through the natural channel.

Dr. Wilkinson conceived the case to have originated in disease of the pubis, which exfoliated and occasioned the successive abscesses, and eventually the communication with the bladder.

A paper was read by Dr. Geary, jun. on a new application of the Ergot of Rye. He stated several cases to prove the advantage of its employment in cases of gonorrhœa.

